# Supplementary figures and images for: Robust quantification of multiplexed fluorescent protein‐based biosensors in plant tissues
Source: Plant J. 2026 Jul 19;127(2):e71034. doi: 10.1111/tpj.71034 (PMC13381040; doi:10.1111/tpj.71034)

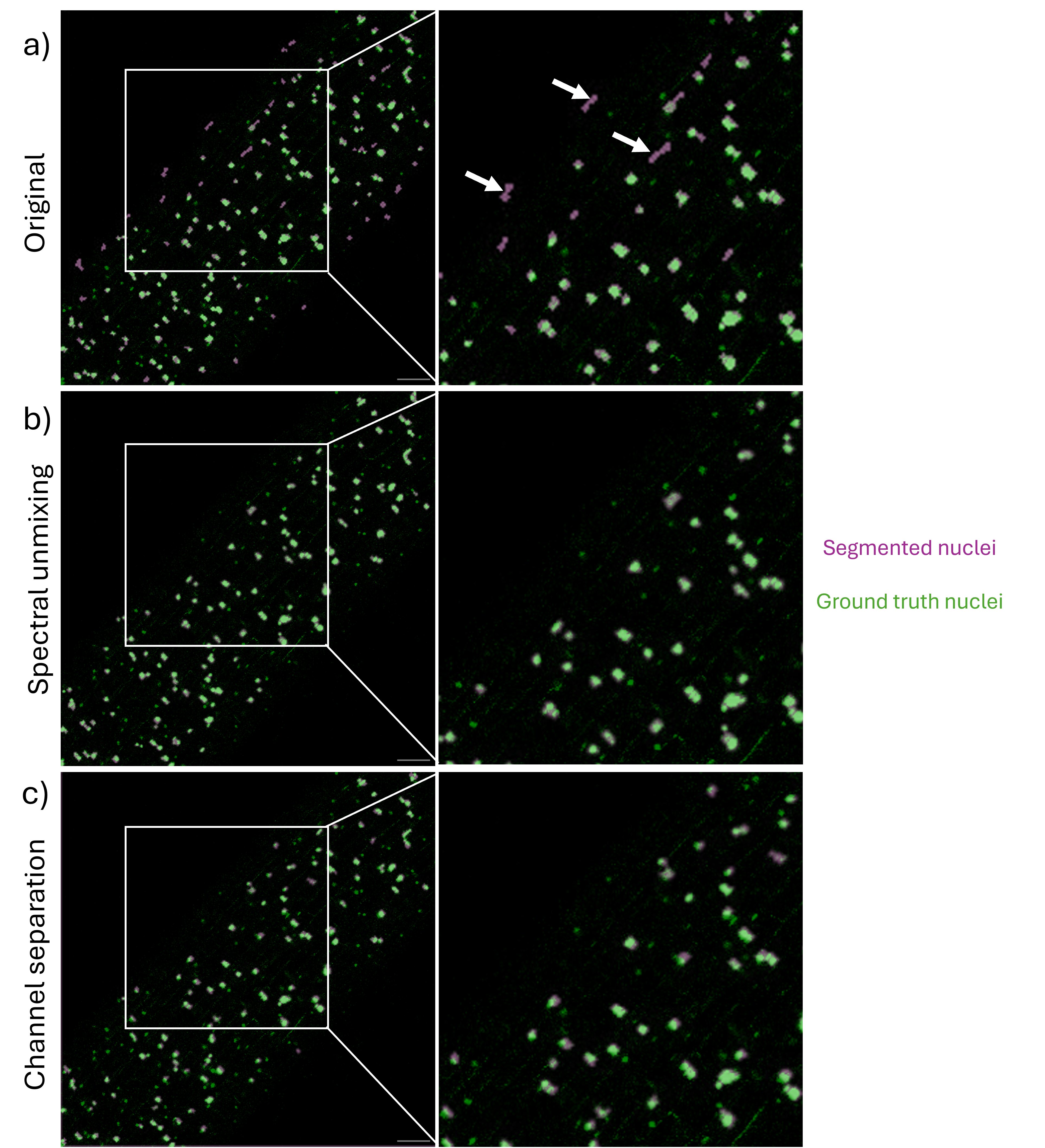

Supplement: Supplementary file 1 — Figure S1. Spectral unmixing and channel separation enable correct segmentation of nuclei in plant root. Ground truth nuclei are marked in green, automatically segmented nuclei in purple. (a) Many nuclei were falsely segmented on image before unmixing (some instances pointed with arrows). Segmentation after (b) spectral unmixing and (c) channel separation is correct. Scale bar: 100 μm. [file TPJ-127-0-s002.tif]

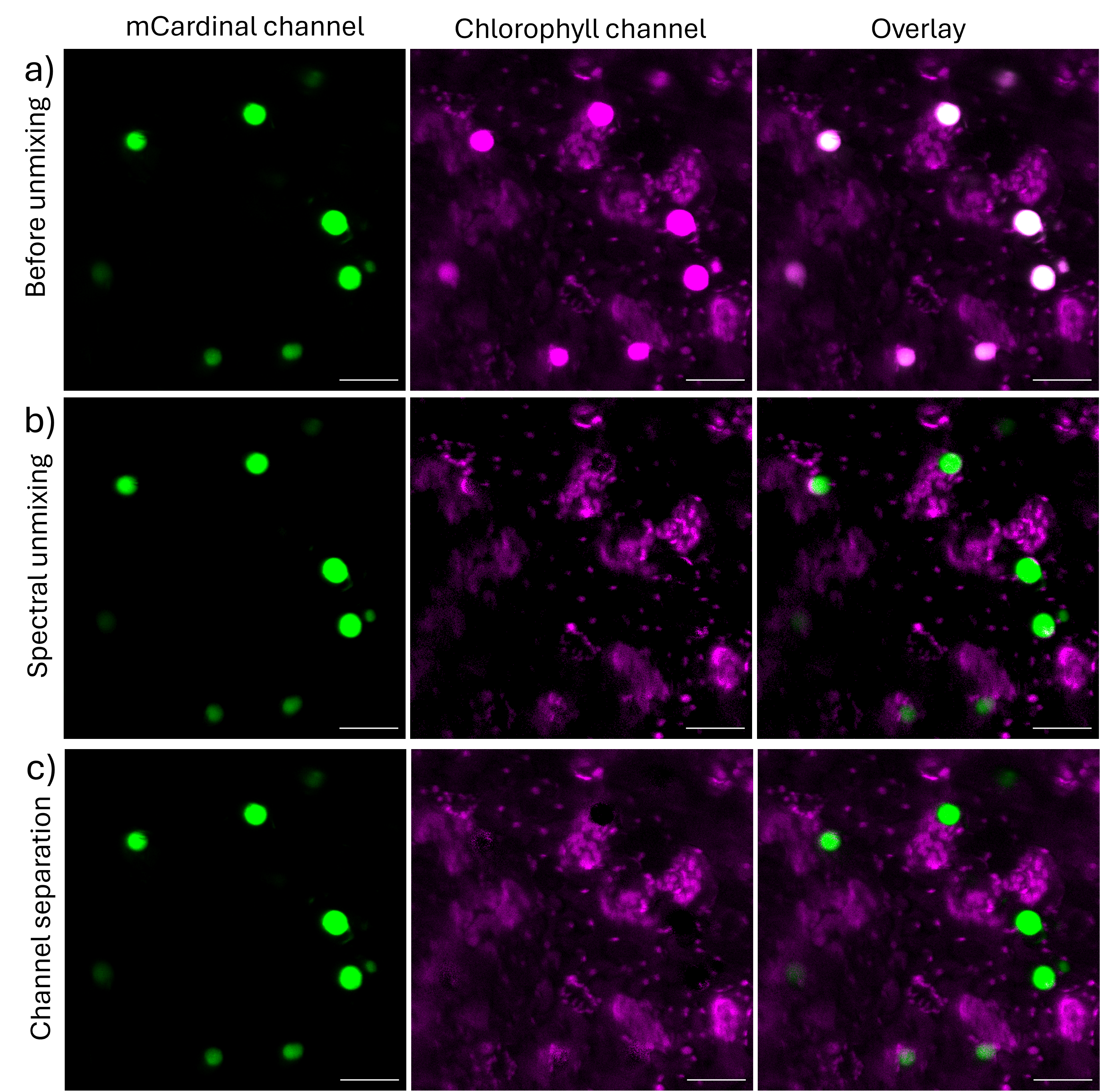

Supplement: Supplementary file 2 — Figure S2. Chlorophyll autofluorescence can be unmixed from mCardinal based on emission spectra. (a) Before unmixing, (b) spectral unmixing using recorded reference spectra, (c) channel separation of mCardinal and chlorophyll. From left to right: mCardinal channel, chlorophyll channel, and overlay of both channels. Scale bar: 50 μm. [file TPJ-127-0-s001.tif]
